# A network approach to the five-facet model of mindfulness

**DOI:** 10.1038/s41598-021-94151-2

**Published:** 2021-07-23

**Authors:** Alexandre Heeren, Séverine Lannoy, Charlotte Coussement, Yorgo Hoebeke, Alice Verschuren, M. Annelise Blanchard, Nadia Chakroun-Baggioni, Pierre Philippot, Fabien Gierski

**Affiliations:** 1 Psychological Sciences Research Institute, Université catholique de Louvain, Louvain-la-Neuve, Belgium; 2 Institute of Neuroscience, Université catholique de Louvain, Brussels, Belgium; 3 Virginia Institute for Psychiatric and Behavioral Genetics, Department of Psychiatry, Virginia Commonwealth University, Richmond, VA USA; 4 Department of Psychiatry and Behavioral Sciences, Stanford University, Palo Alto, CA USA; 5 Laboratoire de Psychologie Sociale et Cognitive, Université Clermont Auvergne, Clermont-Ferrand, France; 6 Department of Psychology, Université de Reims Champagne-Ardenne, Reims, France; 7 INSERM UMR 1247–Research Group on Alcohol & Pharmacodependences, Université de Picardie Jules Verne, Amiens, France

**Keywords:** Psychology, Human behaviour

## Abstract

Despite the large-scale dissemination of mindfulness-based interventions, debates persist about the very nature of mindfulness. To date, one of the dominant views is the five-facet approach, which suggests that mindfulness includes five facets (i.e., Observing, Describing, Nonjudging, Nonreactivity, and Acting with Awareness). However, uncertainty remains regarding the potential interplay between these facets. In this study, we investigated the five-facet model via network analysis in an unselected sample (*n* = 1704). We used two distinct computational network approaches: a Gaussian graphical model (i.e., undirected) and a directed acyclic graph, with each model determining the relations between the facets and their relative importance in the network. Both computational approaches pointed to the facet denoting Acting with Awareness as playing an especially potent role in the network system. Altogether, our findings offer novel data-driven clues for the field's larger quest to ascertain the very foundations of mindfulness.

## Introduction

There has been massive interest in mindfulness over the last three decades^1,2^. Much of this enthusiasm stemmed from the many clinical trials that emphasized the benefits of mindfulness-based interventions (MBIs) for an extensive array of outcomes. Meta-analyses have indeed indicated that MBIs yield medium-sized impacts on mental and physical well-being (e.g., depression relapse, anxiety, chronic pain)^3,4^. Other meta-analyses have likewise reported similar effect sizes on neurocognitive^5^ and social functioning^6^. As a result, governing and political bodies have increasingly given heed to mindfulness, leading to scalable implementations across health, organizational, educational, penitentiary, and military contexts^1,2^.

However, despite escalating interest in the applications of MBIs, uncertainty remains regarding the very nature of mindfulness^2,7,8^. Although mindfulness is typically defined as one’s capacity to pay attention to the present moment with awareness and without judging the inner experience^2,7,8^, research on the nature of mindfulness has been, to date, mostly confined to the development of an operational definition and corresponding reliable measurement tools^9–13^. It is worth noting that most of these definitions refer to mindfulness as a trait-like construct—i.e., reflecting one’s general propensity to be mindful in daily life^9^.

When these operational measures first emerged in the early 2000s, the initial operationalization focused on mindfulness as a unitary construct^12–14^. However, several critical prospects regarding this approach quickly stood out, including the caveat that such a unitary approach thwarts the possibility of distinguishing between specific features of mindfulness, like those developed during MBIs, and their relations with other variables of interest (^10,11,14,15^, for a general discussion on the limitations of relying on single-factor latent approach, see^16^). As a result, several multi-faceted operationalizations of mindfulness arose^9–14^.

Among these conceptualizations, the approach developed by Baer and her colleagues^10,11^ has quickly become a highly disseminated one. To formulate a multi-faceted operational definition of mindfulness, these scholars^10,11^ devised the Five Facet Mindfulness Questionnaire (FFMQ). For the item development of their scale, they drew on previous mindfulness questionnaires and theoretical models representing sets of interdependent skills at play during MBIs^10,15,17–19^, thus representing an amalgamation of various overlapping operational conceptualizations of mindfulness (for a discussion, see^19^). In contrast to unitary approaches to mindfulness, the five-facet approach suggests that mindfulness comprises five facets (i.e., Observing, Describing, Acting with Awareness, Nonreactivity, and Nonjudging), each respectively denoting the dissociable ability to: (1) attend or notice inner (e.g., sensations, thoughts, feelings) and outer (e.g., sights, sounds, smells) experiences; (2) note or mentally label these moment-to-moment experiences with words; (3) bring undivided attention and full awareness to current actions and act with conscious intention (as opposed to running on autopilot without conscious awareness); (4) allow unpleasant thoughts, feelings, and sensations, to come and go, without reacting to them; and (5) approach sensations, cognitions, and emotions without a judgmental attitude.

At the metric level, several studies relying on either exploratory or confirmatory factor analytic approaches have accordingly identified a five-factor solution as the best latent model of the FFMQ’s items across many diverse groups (including people with and without meditation experience), languages, cultures, and contexts (e.g.^11,20–22^). And this five-facet approach to mindfulness has accordingly provided a foundation for studying mindfulness in a multi-dimensional fashion. In this way, it has encouraged examining how the distinct components of mindfulness relate to other psychological constructs, including clinical outcomes such as anxiety and depression symptoms^23,24^, as well as core emotion regulation processes^25^.

Yet, although this multi-faceted approach has undoubtedly opened new vistas in clarifying the connections between specific features of mindfulness and other variables of interest, uncertainty persists regarding the very nature of mindfulness, especially vis-à-vis the potential interplay between its constitutive features—i.e., the facets^20,26^. This is unfortunate, as the five-facet approach was built upon theoretical models assuming sets of *interrelated* skills as the driving force of mindfulness^10,15,17,18,25,27^.

A common view holds that the five facets reflect differential but related constitutive elements of mindfulness^11^. Accordingly, a few studies have reported a hierarchical factor measurement model, with the five facets loading onto a single, assumedly higher-order, latent variable: namely, mindfulness^11,20,21,26^. However, such a perspective assumes that mindfulness functions as an ultimate, overall latent factor that acts as the common latent cause of the five facets. From a metric perspective^28,29^, this can be considered a reflective model of the relationship between the overall latent construct and its related constitutive facets such that here, mindfulness can presumably be viewed as the latent cause of the five facets.

As an alternative to the latent approach, Borsboom and his colleagues have advanced a network approach to psychological constructs^28,29^. In this perspective, these scholars credited the emergence and covariations between the distinct constitutive features of the psychological construct of interest to the direct interactions among the elements themselves^29–34^. In this way, the network approach diverges from the latent variable model, wherein such direct interactions between elements are forbidden given the assumption (axiom) of conditional independence, which assumes that the observed sets of indicators are independent, given the latent construct(s) (for extended discussion on the network approach, see^28–33^). In contrast, the network approach views the psychological construct of interest (i.e., mindfulness) as arising from the interactions of its components, i.e., the facets^28–33^.

Though recent, this network approach has rapidly gained traction in today's psychology research^28–33^. Although the original iteration of the network theory focused mainly on networks comprised solely of psychiatric symptoms, the network approach has since widened to include non-symptom variables (for discussion, see^34^), such as cognitive and emotional processes (e.g.,^35,36^), personality (e.g.,^37,38^), and family-related processes (e.g.^39^). In general, a network approach to psychological concepts discards a common cause explanation and instead views psychological phenomena as interactive system of components (for a discussion and comparison with other approaches, see^29,32,33,37,38^. As such, this view always prioritizes investigations at the component-level instead of focusing on the phenomenon as a whole^31,33,40^, which can be helpful to reconceptualize research and theory for a variety of psychological fields of study^33,40^.

With regard to mindfulness, only two studies thus far have applied a network analytic framework to examine the interdependence between the five facets of mindfulness, and these studies specifically focused on the relations of the facets with other clinical psychological constructs^41,42^. In one study, Medvedev and colleagues^41^ examined the relations between the facets of mindfulness, compassion, positive and negative affect, and psychological distress. They found that Non-judging was inversely related to all aspects of psychological distress, whereas Non-reactivity and Acting with Awareness acted as bridges between protective and maladaptive factors. In the other study, Roca and colleagues^42^ compared the network structure of the relations between the five facets and 20 other variables embracing compassion, psychological well-being, psychological distress, emotion regulation, and attention control before and after an 8-week MBI. They found that, in the network, the connections of the facets of mindfulness with self-compassion and attention control increased from pre to post-MBI. Among the facets, acting with awareness, non-reactivity, and non-judging appeared to play a pivotal role in these changes.

However, these two studies focused on examining the relationships between the five facets with psychological risk and protective factors. None focused on investigating the interdependence between the five facets per se. This is particularly unfortunate, as the five-facet approach was built upon theoretical models, which assumed sets of interrelated skills as the driving force of mindfulness^10,15,17,18,25–27^. Likewise, these two previous studies^41,42^ relied upon partial correlations and did not probe the probabilistic dependencies among the facets. As a result, uncertainty remains regarding whether certain facets are probabilistically more dependent upon the presence of others than vice versa. For instance, following prominent views of mindfulness (e.g.^17^), one may wonder whether activating the node denoting the describing facet probabilistically implies that the ability to bring undivided attention and full awareness to ongoing actions—that is, acting with awareness facet—is already acquired of if the other way around is more likely.

In the study reported here, we thus had two primary goals. First, we endeavored to clarify the pairwise connections among the five facets of mindfulness. To this end, we computed a graphical Gaussian model (GGM) model, an undirected network model wherein the edges represent conditional independent relationships between nodes^43^. Relatedly, we also quantified each node's importance to the resulting network structure via the computation of centrality metrics^43^. Second, we relied on Bayesian network methods to estimate a directed acyclic graph (DAG), which encodes the conditional independence relationships between the variables of interest and characterizes their joint probability distribution^33^. A DAG is a directed network wherein each edge has an arrow tip on one end, indicating the direction of probabilistic dependence (e.g.^33,44,45^). Hence, the resulting network is directed and possesses arrows reflecting the predicted direction of the probabilistic dependence among nodes^33,44^—that is, whether the presence of node X in the network probabilistically implies the presence of node Y (node Y→node X) more than vice versa (node X→node Y)^33,44^. In this project, we thus relied on DAGs to examine the probabilistic dependencies between our variables of interest and generate a data-driven computational model of the interplay among the five facets of mindfulness.

## Results

Following previous applications of network analysis to dispositional psychological constructs (e.g.^36,39,45^), we formed the network at the facet-level instead of the item-level. Descriptive information (i.e., mean, standard deviation, skewness, kurtosis, and range) as well as the Pearson zero-order correlations between the variables are provided in the supplementary materials (see Table S1 and Figure S1).

### The graphical Gaussian model (GGM)

**Figure 1:**
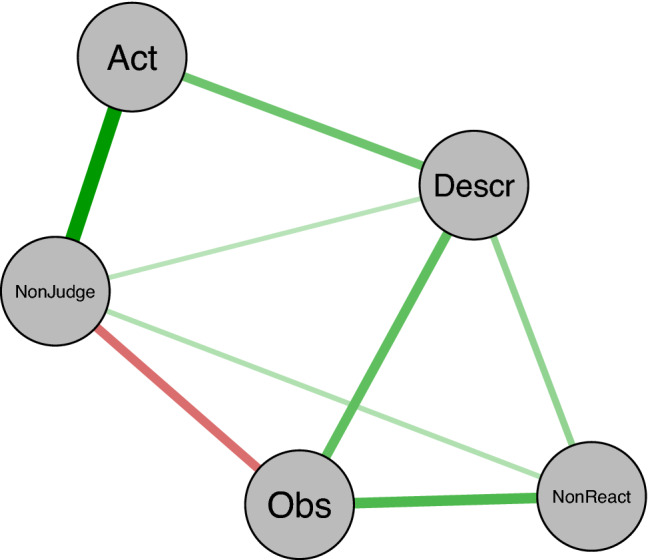
Graphical Gaussian model of the five facets of mindfulness. The strongest edge weight represents a value of 0.38. Green lines reflect positive correlations, whereas red lines reflect negative correlations. *Obs* observing, *Desc* describing, *Act* acting with Awareness, *NonReact* nonreactivity to inner experience, *NonJudge* nonjudgment of inner experience.

Figure 1 depicts the GGM network, which here illustrates the regularized partial correlations between each variable. Green edges characterize regularized positive associations, whereas the red ones signify regularized negative associations. The thickness of the edge denotes the strength of the association, with a thicker edge denoting a larger association. We used the Fruchterman and Reingold’s layout algorithm to determine node placement, so that nodes closer to the center of the network tend to yield the strongest associations with other nodes^43^.

**Figure 2:**
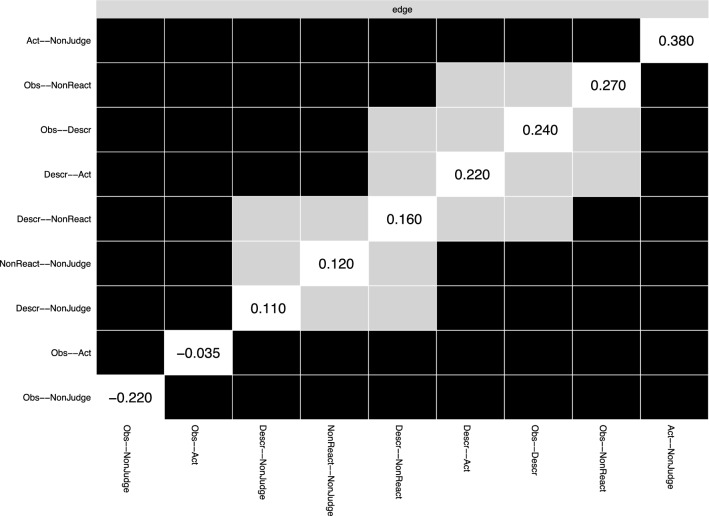
Significant difference between edge weight values. Gray boxes indicate edges that significantly differ between them; black boxes represent edges that do not. Values in the diagonal correspond to the magnitude of the difference, with larger values denoting larger difference. *Obs* observing, *Desc* describing, *Act* acting with awareness, *NonReact* nonreactivity to inner experience, *NonJudge* nonjudgment of inner experience.

A few associations stood out: Nonjudging and Acting with Awareness (*r* = 0.38), Observing and Nonreactivity (*r* = 0.27), Describing and Observing (*r* = 0.24), and Describing and Acting with Awareness (*r* = 0.22). The only negative regularized partial correlation was between Observing and Nonjudging (*r* = − 0.22). We also verified the certainty and precision of the edge weight estimates (see Figure S2 in the Supplementary Materials). Moreover, the bootstrapped difference test indicated that the strongest edges were significantly larger than most others (see Fig. 2).

**Figure 3:**
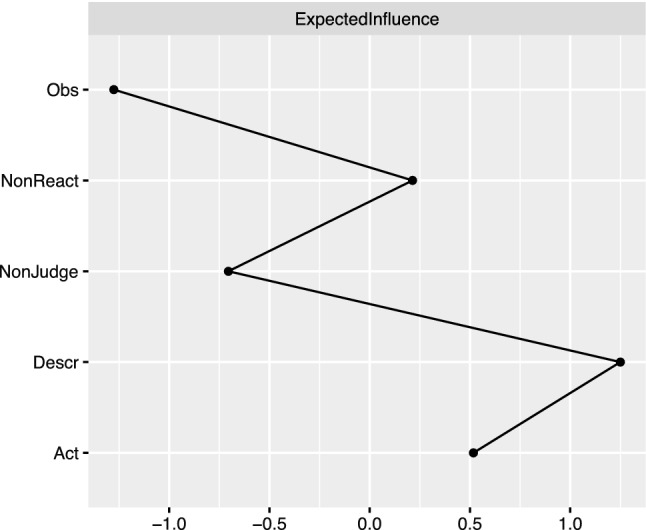
Expected influence estimates of the graphical Gaussian model. *Obs* observing, *Desc* describing, *Act* acting with awareness, *NonReact* nonreactivity to inner experience, *NonJudge* nonjudgment of inner experience.

**Figure 4:**
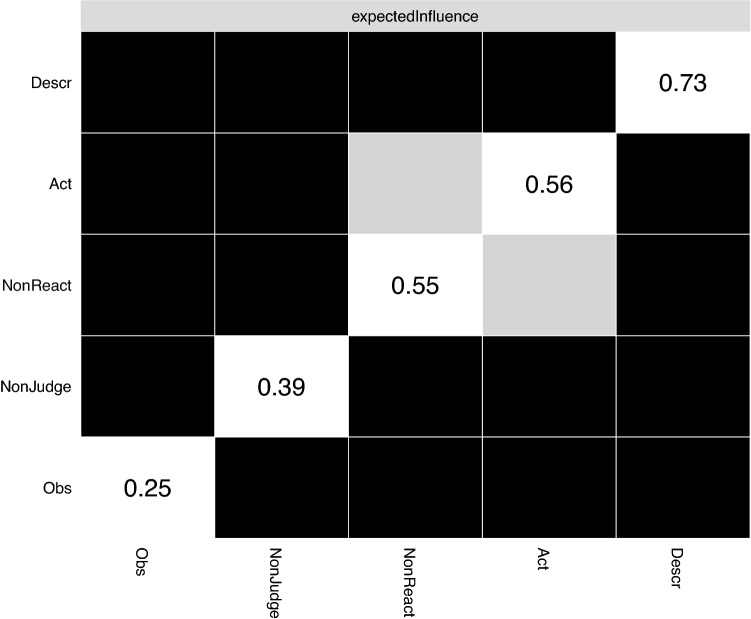
Significant difference between node centrality estimates. Black boxes represent nodes that significantly differ between them in terms of expected influence values; Gray boxes indicate nodes that do not. Values in the diagonal correspond to the magnitude of the difference, with larger values denoting larger difference. *Obs* observing, *Desc* describing, *Act* acting with awareness, *NonReact* nonreactivity to inner experience, *NonJudge* nonjudgment of inner experience.

Figure 3 depicts the expected influence estimates. Higher values signify greater centrality and thus stronger association to other nodes. Describing and Acting with Awareness were the two nodes yielding the highest expected influence values in the GGM. Note that Observing yielded the lowest expected influence value. Moreover, the output of the person-dropping bootstrap approach^43^ revealed that the stability of the centrality estimates was high (see Figure S3 in Supplementary Materials). As shown in Fig. 4, the bootstrapped different test indicates that Acting with Awareness and Describing were significantly more central than most other nodes.

### Directed acyclic graphs (DAGs)

**Figure 5:**
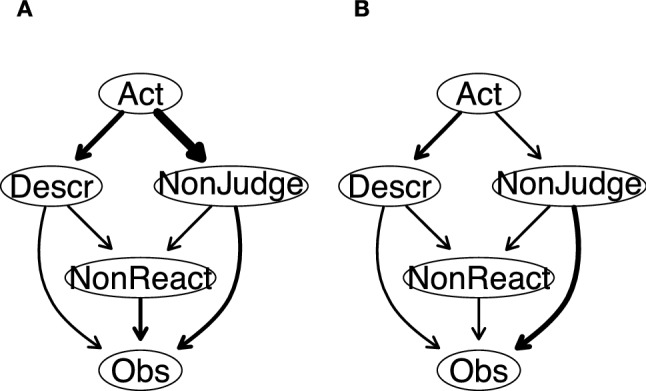
Directed acyclic graphs (DAGs). (**A**) Arrow thickness denotes the importance of that arrow to the overall network model fit. Greater thickness reflects larger contribution to the model fit. (**B**) Arrow thickness indicates directional probability. Greater thickness reflects larger proportions of the bootstrapped networks wherein the arrow pointed in that direction. *Obs* observing, *Desc* describing, *Act* acting with awareness, *NonReact* nonreactivity to inner experience, *NonJudge* nonjudgment of inner experience.

Figure 5 shows the DAGs resulting from 10,000 bootstrapped samples. In both DAGs, arrows that are present in the graph were retained because their strength was greater than the optimal cut-point resulting from the Scutari and Nagarajan method^46^.

In Fig. 5A, arrow thickness denotes the change in the Bayesian Information Criterion (BIC; a relative measure of a model’s goodness-of-fit) when that arrow is removed from the network. In other words, the more an arrow contributes to the model fit, the thicker it is^33,44^. The most important arrows to the network structure connect Acting with Awareness to Nonjudging (with a change in BIC of − 171.58), Acting with Awareness to Describing (with a change in BIC of − 65.23), and Nonreactivity to Observing (with a change in BIC of − 61.23). Table 1 depicts the change in BIC value for each arrow.

**Table 1:** Arrows weight values in the directed acyclic graphs.

Arrow		Value determining arrow thickness	
From	To	BIC	Directional probability
Descr	Obs	− 47.98	0.60
Descr	NonReact	− 50.97	0.57
Acting	Descr	− 65.10	0.72
Acting	NonJudge	− 171.58	0.64
NonReact	Obs	− 61.23	0.64
NonJudge	Obs	− 55.47	0.77
NonJudge	NonReact	− 0.15	0.56

BIC = change in Bayesian Information Criterion when that arrow is removed from the network. BIC values determine arrow thickness in Fig. 5A (reflecting the importance of that edge to the network structure). For the BIC values, negative values correspond to decreases in the network score that would be caused by the arrow’s removal. In other words, negative scores mean that model fit improves with the presence of that arrow. Directional probability values determine arrow thickness in Fig. 5B (reflecting the frequency that arrow was present in that direction in the 10,000 bootstrapped networks).

*Obs* observing, *Descr* describing, *Acting* acting with awareness, *NonReact* nonreactivity to inner experience, *NonJudge* nonjudgment of inner experience.

In Fig. 5B, the thickness of the arrows represents directional probabilities—that is, the proportion of the averaged 10,000 bootstrapped networks wherein that arrow was pointing in that direction. The thickest arrow connects Acting with Awareness to Describing (with a directional probability of 0.72; i.e., this edge was pointing in that direction in 7200 of 10,000 bootstrapped networks, and in the other direction in only 2800 of the bootstrapped networks). The thinnest arrows connect Nonjudging to Nonreactivity (with a directional probability of 0.56) and Describing to Nonreactivity (0.57). The directional probability for each arrow in Fig. 5B can be found in Table 1.

Structurally, because DAGs encode the conditional independence relationships and portray the joint probability distribution of each node, the organization of a node within a DAG can be seen as a product of each node's conditional distribution knowing its parent nodes in the estimated model^33,39,44^. Here, because Acting with Awareness emerged as the parent node in the model, it implies that Describing is likely to be probabilistically dependent on it, thus suggesting that participants are more prone to report elevated scores on the Describing facet if they report elevated score on the Acting with Awareness facet than the other way around. Likewise, Nonjudging was also probabilistically dependent on Acting with Awareness. In turn, Nonreactivity was probabilistically dependent on both Describing and Nonjudging. Finally, Observing appeared at the bottom of the probabilistic cascade.

## Discussion

There has been considerable enthusiasm over the development of operational definitions of mindfulness over the last two decades. Among these definitions, the five-facet approach has quickly become one of the most prominent ones^10,11^. However, this approach considers the construct of mindfulness as the underlying cause of the five facets that supposedly denote mindfulness^11,20,21,28^, thus implying a reflective model of the relationship between mindfulness and its facets, wherein the former presumably causes the latter^28–33^. In contrast, a network approach to psychological constructs has recently appeared^28–33^. When embracing this perspective, mindfulness can be viewed as a formative construct resulting from the interrelationships between its constitutive facets instead of being a presumably latent construct underlying its facets. In this study, we empirically investigated the five-facet approach to mindfulness from this network perspective. To do so, we used two distinct computational network approaches: a GGM and a DAG. In line with the network approach, our results indicate that the five facets can be viewed, within the framework of the network approach, as a set of interacting nodes.

Among the most remarkable finding was the convergence across the distinct computational approaches, despite their varying assumptions and constraints. Indeed, both the GGM and the DAG pointed to Acting with Awareness and Describing as the two facets playing especially crucial roles in the network system. First, the two nodes appeared as highly interconnected in the GGM, and they were incident to the thickest and most vital edges in the network (e.g., the edges connecting Acting with Awareness and NonJudging; Describing and Observing; Describing and Nonreactivity). Second, when considering the centrality estimates, which provide a fine-grained analysis about which nodes are essential to maintaining the network's coherence as a system, Acting with Awareness and Describing both emerged as the two facets yielding the highest centrality. Finally, the DAG elucidated the especially intriguing interrelationship between those two nodes. Acting with Awareness topped the cascading network of probabilistic dependencies between nodes, whereas Describing emerged as probabilistically dependent on it. Because Acting with Awareness emerged at the top of the model, it suggests that, from a probabilistic perspective, someone is unlikely to manifest the distinct mindfulness facets unless they can be aware of their situation and act with conscious intention, rather than react automatically. However, because Observing and Nonreactivity were directly probabilistically dependent on Describing but not directly on Acting with Awareness, our results suggest that Acting with Awareness exerts its influence on Nonreactivity and Observing via Describing and Nonjudging.

The observation that Acting with Awareness emerges as the driving force of mindfulness should not come as a surprise. Instead, it dovetails with prominent views of mindfulness^15,17–19,45^, wherein the ability to bring undivided attention and full awareness to ongoing actions (rather than running on autopilot without conscious awareness) is conceived as the heart of mindfulness. Although the observation that the Describing facet was strongly associated with other mindfulness facets is in line with previous research^11,24,47^, the DAG’s suggestion that Acting with Awareness may constitute the driving force of the entire mindfulness’ network system fully aligns with Kabat-Zinn's initial conceptualization of mindfulness^17,48^, centering on deliberately attending to activities and experiences from the present moment rather than describing or labeling these experience with words. Moreover, several longitudinal studies conducted during MBIs pointed to Acting with Awareness as a facet of mindfulness that predicted beneficial improvement in mindfulness and mental health over time (e.g.^23,49,50^). Although our cross-sectional design precludes any strong inference regarding the temporal dynamics of the mindfulness facets per se, the results invite the hypothesis that Acting with Awareness might be an especially potent constituting feature of mindfulness.

Of note, like the two previous network studies comprising the five facets^41,42^, there was no direct association between Acting with Awareness and Non-Reactivity. As suggested Medvedev and colleagues^41^, these two facets might act as bridges between the facets and maladaptive factors such as depression and negative affect, by prompting responding to external and internal stressors through acting with awareness and allowing unpleasant thoughts, feelings, and sensations to come and go without reacting to them. A future step will thus be to capitalize on temporal network analyses from intensive longitudinal research designs to further elucidate the role of these two facets.

Another striking finding was our observation that the Observing facet yielded the lowest centrality values and was tied to the only negative edge (along with Nonjudging). This finding fully aligns with previous research suggesting that this facet might play a distinct role^51^. Of note, the presence of a negative association between Observing and Nonjudging aligns with a similar observation in previous network research including the FFMQ^41^. Because research has suggested that this facet may only yield beneficial influence in meditating samples^11,21,41^, a critical next step would thus be to compare the network structure and node centrality between meditating and non-meditating samples.

The present study may have relevant practical implications. Network models posit that central nodes may play a pivotal role in the maintenance of the network system^28–36^. From this perspective, activating a highly central node (i.e., a higher score on this facet) would trigger other nodes via direct and indirect paths, thereby engendering a cascade of node activation^33^. Moreover, because the DAG reveals that the different facets are less likely to be activated or to sustain one another in the absence of Acting with Awareness, our results also suggest that this facet may constitute an early hint toward the acquisition of mindfulness-related skills, deserving an especially careful audit during MBIs. And it bears repeating that this suggestion fully dovetails with Kabat-Zinn's initial conceptualization of mindfulness^17,48^, which focused on deliberately attending to the present moment during daily activities and experiences as the primary component of mindfulness' practice.

A critical next step will thus be to examine how the network structure of the five facets, and especially the highly central role of Acting with Awareness, is impacted by distinct types of meditation practices. This issue is especially important given that the FFMQ is indebted to the Dialectical Behavior Therapy (DBT) approach^10,11,47^, and one may thus wonder whether other types of MBI have an equal impact on the network structure of the five facets. On the other hand, numerous studies indicated that distinct non-DBT-based MBIs do impact FFMQ scores and subscores (e.g.^47^). Another capstone in future iterations would be to examine how the network structure of the five facets is impacted by the experimental manipulation (or training) of different cognitive functions assumedly involved in the emergence of dispositional mindfulness. Given the recent theoretical emphasis on one’s metacognitive beliefs about self-regulatory processes to explain how mindfulness may arise in everyday life (e.g.^52,53^), future iterations may thus want to capitalized on these processes when considering the potential between- and within-person fluctuations of the network structure of the five facets of mindfulness.

The present study has several limitations that require further investigations in future studies. First, we focused only on trait mindfulness, precluding any inference from our findings to state mindfulness. A key step in future iterations will thus be to examine the network structure of state mindfulness. Moreover, following the seminal work of Fridhandler^54^ on the distinction between psychological traits and states as being respectively best construed as dispositional and occurrent concepts, a capstone would be to explore whether individuals with high trait mindfulness (and especially those scoring high on Acting with Awareness) are more prone to experiencing frequent, intense, and prolonged episodes of state mindfulness than those low on trait mindfulness.

Second, the estimation of both the GGM and DAGs relies on cross-sectional data, thus excluding any strong inference regarding the potential causal relations between the five facets. The only insight into the possible direction of associations is from the DAG, which uses probabilistic Bayesian learning methods to provide clues about this direction^33^. Yet, when considering DAGs based on cross-sectional data, one must not confuse the direction of dependence with temporal antecedence^33,36^. DAGs provide clues about probabilistic dependencies between the variables and should be used in a hypothesis-generating fashion rather than a hypothesis-testing one (for discussion, see^33,36^). DAGs thus do not signify the temporal precedence of the top-cascading node in the DAG^33^.

Third, DAGs assume, by definition, that connections between nodes are directed and acyclic. Yet, relationships between variables cannot always be defined as directed and acyclic relations of probabilistic dependencies (e.g., in the case of feedback loops). However, because the direction of the arrow is determined by the percentage of bootstrapped networks wherein this arrow was pointing in that direction, the degree of potential reverse directionality can be estimated from the proportion of bootstrapped networks wherein the arrow pointed in the other direction^33,44^. Here, a few arrows were relatively thin, indicating that the direction of the arrow was pointing in the other direction in a substantial proportion of the bootstrapped networks^34,36,39^. For instance, the edge connecting NonJudging to NonReacting pointed in that direction in 56% of the 10,000 bootstrapped networks, thus implying that it pointed in the other way in 44% of the 10,000 bootstrapped networks (see Table 1). The direction of the association between these two facets may thus tip in both directions. Elucidating the potential bidirectional dependencies between variables would require the application of temporal network analyses on data arising from experience sampling methods.

Fourth, some commentators have questioned the suitability of centrality indices, partly because some have appeared unstable in cross-sectional and temporal networks (e.g.^40,55^). However, most of these concerns focused on closeness and betweenness centrality metrics and not expected influence, which was the metric used here. Indeed, this latter has been typically reported as a more stable metric^56^; an observation confirmed in this study, as expected influence estimates were highly stable (see Supplementary materials).

Finally, although some studies have suggested the highly predictive nature of especially central nodes in determining the onset, course, and recovery of psychological disorders (for an in-depth discussion, see^33^), one may question the relevance of considering central nodes as valuable therapeutic targets. It might indeed be challenging to target a highly central node without immediately affecting the other nodes^57^.

In conclusion, these limitations notwithstanding, the present results confirm the plausibility of considering the five-facet model of mindfulness as a network system wherein the facets are viewed as interacting nodes. As such, our data-driven approach thus paves new ways for the quest to ascertain the very foundations of mindfulness.

## Methods

### Preregistration and open science practices

The study design, data collection, and analysis plan were preregistered at https://osf.io/ycnp7. Our R code and de-identified data are available at https://osf.io/qtkzm/.

Note that we had three minor deviations from our preregistration. First, we included more participants than described in the preregistration. Whereas our target sample size was 650 participants, 1764 participants completed the survey. We decided to include all participants rather than selecting a subsample of our dataset. Second, to align with prior research, we also deviated by using the sum score of the facets instead of the mean score (i.e., sum score divided by the number of items, as described in the preregistration). However, after running the analyses, we recognized that both computations generated the exact same network models. For ease of interpretation, we decided to rely on the sum scores. Finally, we initially planned to examine the community structure of the five facets, that is, to test whether the five nodes of the GGM cluster into one or multiple communities. However, we realized that optimally achieving this goal would require an item-level network (i.e., each of the 39 FFMQ-items as a node in the GGM) instead of a facet-level network^58^. In addition, our focus was on the interdependence between the theory-driven five facets of trait mindfulness, and particularly their conditional independence relationships via the estimation of DAGs. We thus dispensed with the community detection approach and kept our analysis at the facet-level.

### Participants

We recruited 1764 French-speaking participants (57.40% women). They were recruited from the general community through listserv advertisements and local media. Participants had no experience of mindfulness practice. Within the sample, 48.58% (*n* = 857) were from Belgium and 51.44% (*n* = 907) from France. Participants were between 17 and 81 years old (*M* = 25.69, *SD* = 12.19). Following previous studies (e.g.^32–34^), we removed participants with missing values (*n* = 60) and only kept those who had completed all items. The analyses were thus performed on the remaining 1704 participants. The study received the approval of the local review board (i.e., the biomedical ethical committee of UCLouvain, Belgium, and the ethical committee of Université de Reims Champagne-Ardenne, France) and was carried out following the Declaration of Helsinki. Each participant gave their written informed consent prior to participating in the study.

### Measures

The FFMQ^11^ is a 39-item scale that measures trait mindfulness. It is one of the most commonly-used tools to measure mindfulness. The FFMQ includes five subscales, each assessing one of the five facets: (a) eight items measure the Observing facet*;* (b) eight items measure the Describing facet; (c) eight items measure the Acting with Awareness facet*;* (d) eight items measure the Nonjudging facet*,* and (e) seven items measure the Nonreactivity facet. Participants rate each item on a 5-point Likert-type scale, ranging from 1 (*Never or very rarely true*) to 5 (*Very often or always true*). Items denoting the absence of mindfulness were reverse scored. For each facet, higher scores indicate greater endorsement of that facet.

The FFMQ and each of its facet-related subscales show extremely high reliability and validity (for a review, see^24^). In this study, we relied on the validated French-speaking version of the scale^21^. The Cronbach’s alphas were high (0.96 for the global scale) as well as for each of the five subscales (0.88 for Nonjudging; 0.86 for Acting with Awareness; 0.84 for Describing; 0.77 for Observing; 0.77 for Nonreactivity).

### Data analytic approach

#### Check for potential nodes redundancy

Because some of our variables may overlap conceptually (e.g., Nonreactivity and Nonjudging facets), we implemented a data-driven method to confirm that none of our five variables (i.e., the five facets) were redundant. To do so, we followed the procedure described in recent publications (for details, see^35,39^). We first tested whether our correlation matrix was positive definite. Had a non-positive definite matrix emerged, this would reflect that our variables were a linear combination of other variables. We then implemented the Hittner method to search for potential highly inter-correlated (r > 0.50) pairs of variables that also correlated to the same degree with other variables (i.e., > 75% of correlations with other variables did not significantly differ for a given pair). The Hittner method was implemented via the goldbricker function of the R package *networktools*. This method did not identify any redundancy between our five variables.

#### Estimation of the graphical Gaussian model

We present a GGM that was regularized through the graphical LASSO algorithm, which has two main goals^33,43^. First, it estimated regularized partial correlations between pairs of nodes, thereby excluding spurious associations (or edges) resulting from the influence of other nodes in the network. Second, it shrunk trivially small associations to zero, thus eliminating possibly "false positive" edges from the model and returning a sparser network including only the strongest edges. We did so via the R package *qgraph*, which automatically implements such a regularization along with model selection based on the Extended Bayesian Information Criterion (EBIC). This procedure computes 100 models with varying degrees of sparsity; a final model is chosen according to the lowest EBIC value, given a specific hyperparameter gamma (γ), which regulates the trade-off between admitting false-positive edges and suppressing true edges. In general, the hyperparameter γ is set between 0 (favoring a model with more edges) and 0.5 (promoting a simpler model with fewer edges). Following recommendations based on stimulation studies (for details, see^43^), we set γ to 0.5 to be confident that our edges are true. To assess the stability of our edge weights, we implemented a nonparametric bootstrapping procedure (with 1000 bootstrapped samples with replacement) to bootstrap the edge weights' confidence regions. Using a bootstrapped difference test^43^, we also examined whether the edge weights significantly differed from one another.

#### Estimation of the centrality metrics

We estimated the expected influence centrality indices to gauge each node's importance in the regularized GGM^56^. This index quantifies the cumulative importance of each node and describes the sum of the edge weights attached to this node, considering both positive and negative values^56^. Hence, higher values indicate greater centrality in the network and so greater importance. The plot represents the raw expected influence value of each node. Following recent guidelines^43^, we assessed the stability of this metric's estimates by implementing a person-dropping bootstrap procedure (with 1000 bootstrapped samples with replacement) and determined the CS-coefficient (details are available in the supplementary materials). Capitalizing on this person-dropping bootstrap procedure, we performed a bootstrapped difference test^43^ to examine whether nodes significantly differ from one another in terms of centrality estimates.

#### Directed acyclic graph (DAG)

Following prior research (e.g.^36,39,44^), we estimated the DAGs via the implementation of a Bayesian hill-climbing algorithm^46^. To do so, we relied on the R package *bnlearn*. As implemented in this package, this approach relies on a bootstrap function that estimates the structural features of the model by adding edges, removing them, and reversing their direction to ultimately optimize the goodness-of-fit target score, i.e., the Bayesian Information Criterion (BIC; a relative measure of a model’s goodness-of-fit). This bootstrap function requires an iterative procedure of randomly restarting this process with various possible edges connecting various node pairs, disturbing the network system, and applying 50 different random restarts to circumvent local maxima. As in recent implementations of this algorithm (e.g.^36,39,44^), we introduced, for each restart, 100 perturbations (i.e., attempts to insert, delete, or reverse an edge). As this iterative process of restart/perturbations unfolds, the algorithm returns the model with the optimal BIC value.

In keeping with recent publications (e.g.^36,39,44^), we then ensured the stability of the resulting DAG as follows. We bootstrapped 10,000 samples (with replacement), estimated a network for each of the bootstrapped 10,000 samples, and ultimately averaged the resulting 100,000 networks to generate a final network structure via a two-step method. First, we determined how frequently a given edge appeared in the 10,000 bootstrapped networks. We then applied the optimal cut-point approach of Scutari and Nagarajan^46^ for retaining edges, which yields networks with both high sensitivity and high specificity. Second, we determined the direction of each surviving edge in the bootstrapped networks. If an edge pointed from node X to node Y in at least 51% of the bootstrapped networks, then this direction was reported in the final DAG using an arrow pointing from node X to node Y.

For ease of interpretation, we followed prior research^36,39,44^ and produced two visualizations of the resulting outputs. In the first one, the thickness of the arrow represent the change in the BIC values when that arrow is removed from the network. In this way, the thicker the arrow, the more that arrow contributes to the model structure^33,44^. In the second visualization, the thickness of the arrow denotes directional probabilities—that is, the proportion of the bootstrapped networks wherein that arrow was pointing in that direction. In this way, the thicker the arrow, the larger the proportion of bootstrapped networks wherein this edge pointed in the direction depicted^33,44^.

## Supplementary Information

Supplementary Information.
