# Supplementary material for: A network approach to the five-facet model of mindfulness
Source: Sci Rep. 2021 Jul 23;11:15094. doi: 10.1038/s41598-021-94151-2 (PMC8302557; doi:10.1038/s41598-021-94151-2)

Supplementary materials

A Network Approach to the Five-Facet Model of Mindfulness: Insights from Gaussian Graphical and Directed Acyclic Graph Models

Alexandre Heeren*[,1,2], Séverine Lannoy[3,4], Charlotte Coussement[1], Yorgo Hoebeke[1], Alice Verschuren[1], M. Annelise Blanchard[1], Nadia Chakroun-Baggioni[5], Pierre Philippot[1], & Fabien Gierski[3,6]

[1] Psychological Sciences Research Institute, Université catholique de Louvain, Louvain-la-Neuve, Belgium

[2] Institute of Neuroscience, Université catholique de Louvain, Brussels, Belgium

[3] Virginia Institute for Psychiatric and Behavioral Genetics, Department of Psychiatry, Virginia Commonwealth University Richmond, VA USA.

[4] Department of Psychiatry and Behavioral Sciences, Sandford University, Palo Alto, CA, USA

[5] Université Clermont Auvergne, Université Blaise Pascal, Laboratoire de Psychologie Sociale et Cognitive, BP 10448, F-63000 Clermont-Ferrand, France

[6] Department of Psychology, Université de Reims Champagne-Ardenne, Reims, France

[7] INSERM UMR 1247 – Research Group on Alcohol & Pharmacodependences (GRAP), Université de Picardie Jules Verne, Amiens, France

Additional Descriptions of the Variables

Mean, standard deviation, skewness, and kurtosis are presented separately for each node in *Table S1*. The Pearson product-moment correlations between each pair of variables are plotted in *Figure S1*.

Accuracy of the Edge Weights

With the R package *bootnet* (Epskamp, Borsboom, & Fried, 2018), we bootstrapped confidence regions of the edge weights by using a non-parametric approach and sampling data with 1,000 replacements to estimate the accuracy of the graphical LASSO network. The edges were reasonably stable, and 80% of edges exhibited values significantly different than zero (see *Figure S2)*.

Stability of the Centrality and Bridge Centrality Metrics

We then evaluated the stability of the centrality metrics by implementing a subset bootstrap procedure (Costenbader & Valente, 2003). To do so, we repeatedly correlated the centrality metrics of the original dataset with the metrics calculated from a subsample of participants missing via person-dropping bootstraps as implemented in the R package *bootnet* (Epskamp et al., 2018). If correlation values decline substantially as participants are removed, then this centrality index would be considered less stable. We set the bootstraps to 1000. Results indicated that both expected influence and bridge expected influences estimated are highly stable (see *Figure S3).* We also calculated the centrality stability correlation coefficient (CS-coefficient) to quantify the effects of this person-dropping procedure. The CS-coefficient represents the maximum proportion of participants that can be dropped while maintaining 95% probability that the correlation between centrality metrics from

the full data set and the subset data are at least .70. Based on a simulation study (Epskamp et al., 2018), a minimum CS-coefficient of .25 (and preferably ≥ .50) is recommended for interpreting centrality indices. In the present dataset, the CS-coefficients were .75 for the expected influence.

Differential Variability

Recent commentators have argued that differential variability—the phenomenon that variables have drastically different variances—may distort conclusions about node centrality (e.g., Fried, 2016; Terluin, de Boer, & de Vet, 2016). We thus computed the correlations between the standard deviation and the centrality estimates of the five variables to test whether differences in variances may have distorted conclusions about expected influence estimates. The two-tailed Pearson correlation between the standard deviation and expected influence centrality, $r(3) = -.11$, $p = .86$, was not significant. Had a significant correlation emerged, this would suggest that a node's importance in the network was affected by its variability. Taken together, these data suggest the differential variability across variables does not pose a problem for interpreting expected influence estimates in this study.

References

Costenbader, E., & Valente, T. W. (2003). The stability of centrality measures when networks are sampled. *Social Networks, 25*, 283–307.

Epskamp, S., Borsboom, D., & Fried, E. I. (2018). Estimating psychological networks and their accuracy: A tutorial paper. *Behavior Research Methods, 50*, 195–212.

Fried, E. I. (2016, November 26). New paper: differential variability of variables drives network structure [Web log post]. Retrieved from https://psych-networks.com/new-paper-differential-variability-items-drives-network-structure/

Terluin, B., de Boer, M. R., & de Vet, H. C. W. (2016). Differences in connection strength between mental symptoms might be explained by differences in variance: Reanalysis of network data did not confirm staging. *PloS ONE* *11*(11): e0155205

Table S1. Mean (M), standard deviation (SD), minimum (Min), maximum (Max), skewness, and kurtosis of each node.

**Node**	**M**	**SD**	**Min**	**Max**	**Skewness**	**Kurtosis**
Observing	25.68	5.97	8	40	- .22	-.18
Describing	24.97	6.32	8	40	- .03	- .48
Acting with Awareness	27.02	6.37	8	40	- .28	- .29
Nonreacting	19.42	4.94	7	35	- .11	- .05
Nonjudging	27.06	7.07	8	40	- .27	- .53

Figure S1. Pearson product-moment correlations between each facet.

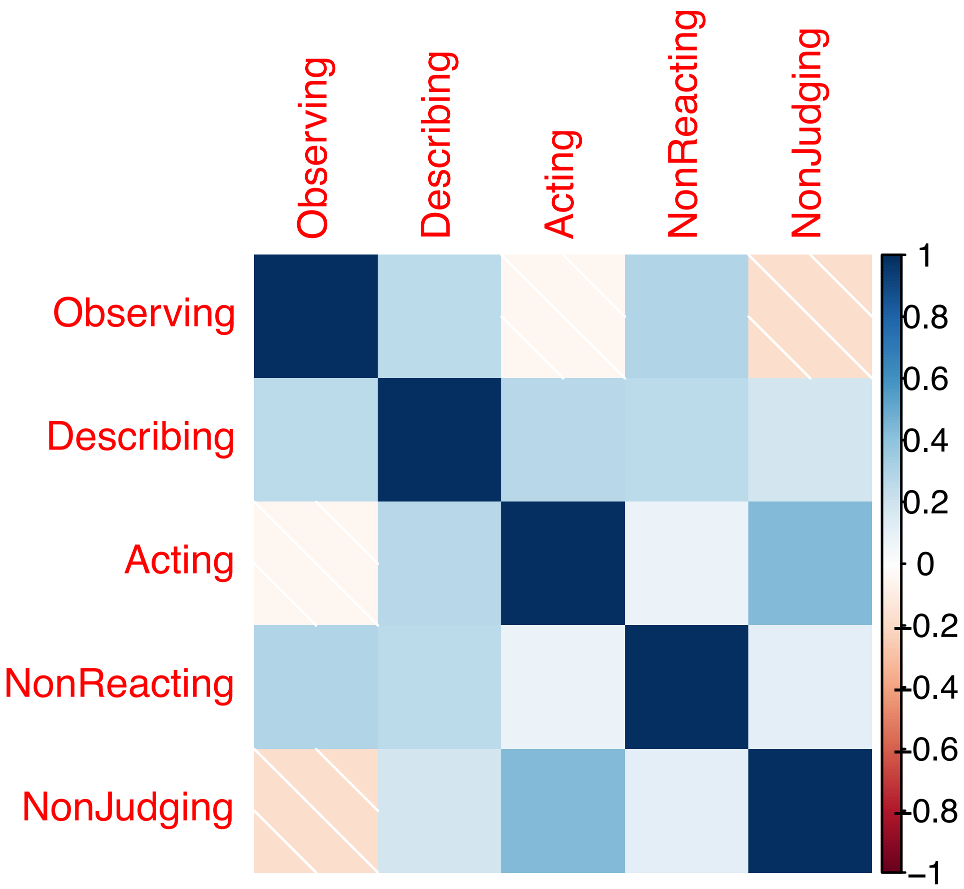

Figure S2. Bootstrapped confidence intervals of estimated edge weights for the graphical lasso network. The red line indicates the sample values. The dark line indicates the bootstrapped mean values.

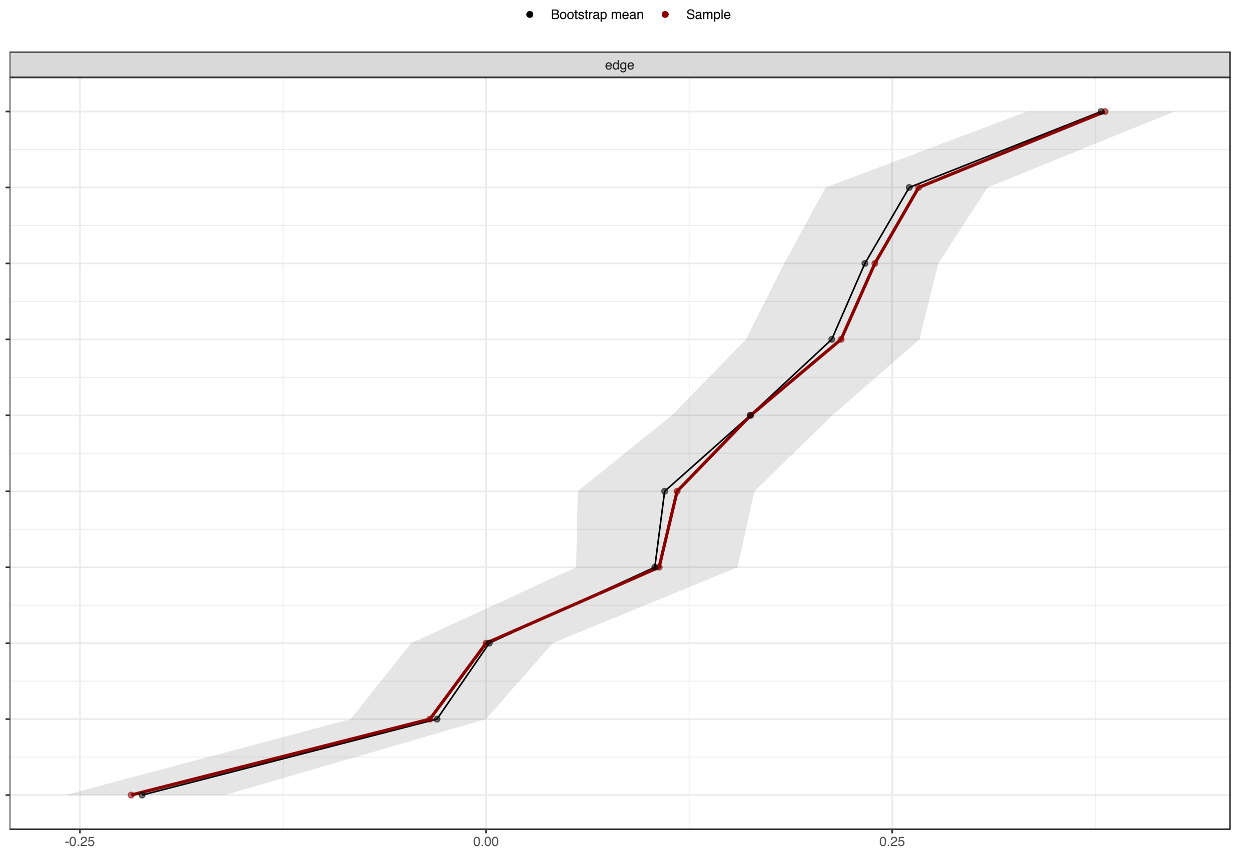

Figure S3. Average correlation between centrality indices (i.e., expected influence) of the network estimation sampled with persons dropped and the original sample.

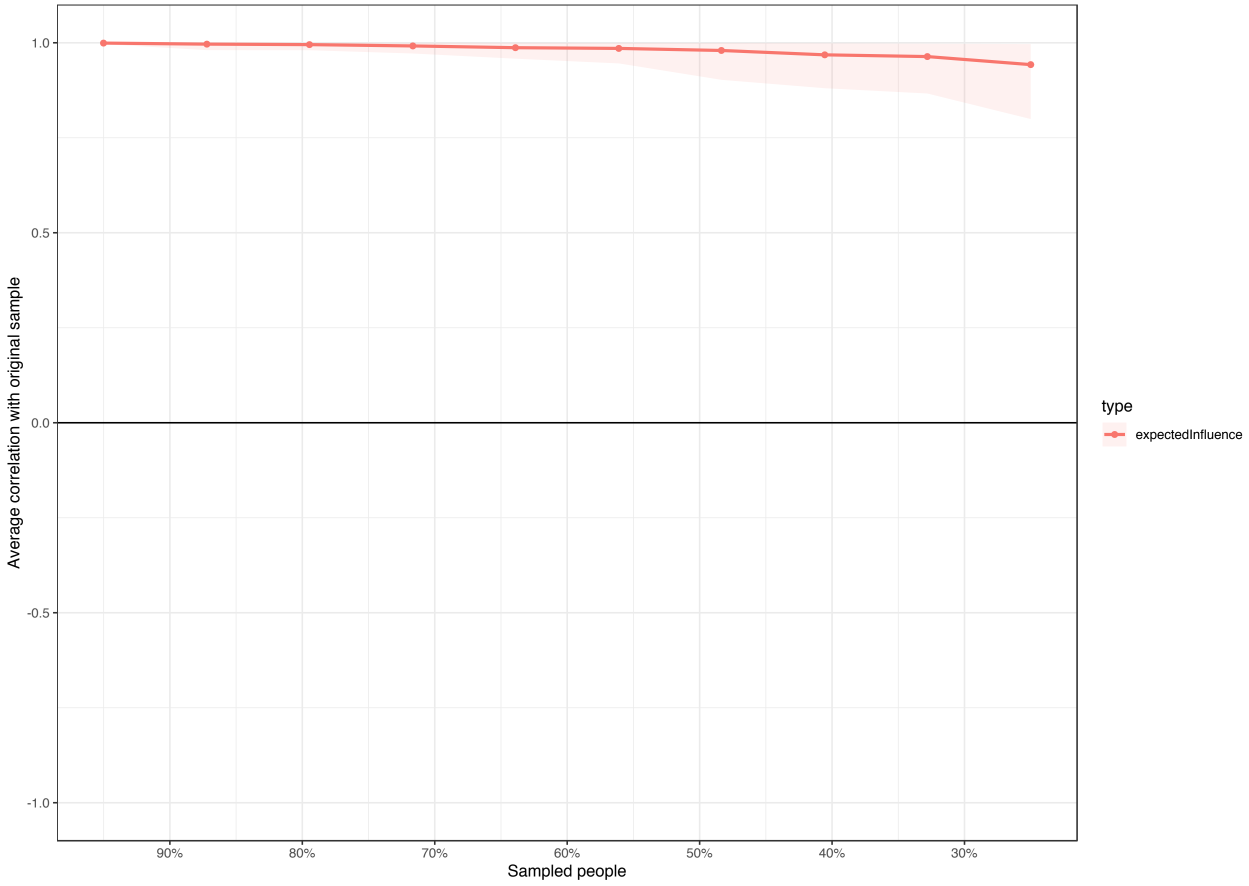

Supplement: Supplementary file 1 — Supplementary Information. [file 41598_2021_94151_MOESM1_ESM.pdf]
